# The Study of Ketamine for Youth Depression (SKY-D): study protocol for a randomised controlled trial of low-dose ketamine for young people with major depressive disorder

**DOI:** 10.1186/s13063-023-07631-3

**Published:** 2023-10-24

**Authors:** Orli S. Schwartz, Paul Amminger, Bernard T. Baune, Gillinder Bedi, Michael Berk, Sue M. Cotton, Rothanthi Daglas-Georgiou, Nick Glozier, Ben Harrison, Daniel F. Hermens, Emma Jennings, Jim Lagopoulos, Colleen Loo, Sumudu Mallawaarachchi, Donel Martin, Bethany Phelan, Nikki Read, Anthony Rodgers, Lianne Schmaal, Andrew A. Somogyi, Lily Thurston, Amber Weller, Christopher G. Davey

**Affiliations:** 1 Department of Psychiatry, University of Melbourne, Melbourne, Australia; 2 Orygen, Melbourne, Australia; 3 Centre for Youth Mental Health, University of Melbourne, Melbourne, Australia; 4 Department of Psychiatry, University of Münster, Münster, Germany; 5 The Florey Institute of Neuroscience and Mental Health, Melbourne, Australia; 6 Deakin University, IMPACT, The Institute for Mental and Physical Health and Clinical Translation, School of Medicine, Geelong, Australia; 7 Central Clinical School, Faculty of Medicine and Health, The University of Sydney, Sydney, Australia; 8 Australian Research Council Centre of Excellence for Children and Families Over the Life Course, Sydney, Australia; 9 Professor Marie Bashir Centre, Royal Prince Alfred Hospital, Sydney, Australia; 10 Thompson Institute, University of the Sunshine Coast, Sunshine Coast, Australia; 11 Thompson Brain and Mind Healthcare, Sunshine Coast, Australia; 12 School of Psychiatry, University of New South Wales, Sydney, Australia; 13 Black Dog Institute, Sydney, Australia; 14 School of Clinical Medicine, University of New South Wales, Sydney, Australia; 15 The George Institute for Global Health, University of New South Wales, Sydney, Australia; 16 School of Biomedicine, University of Adelaide, Adelaide, Australia; 17 Victorian Department of Health, Melbourne, Australia

**Keywords:** Ketamine, Depression, Major depressive disorder, Young people, Youth, Adolescence, Mental Health, Psychiatry

## Abstract

**Background:**

Existing treatments for young people with severe depression have limited effectiveness. The aim of the Study of Ketamine for Youth Depression (SKY-D) trial is to determine whether a 4-week course of low-dose subcutaneous ketamine is an effective adjunct to treatment-as-usual in young people with major depressive disorder (MDD).

**Methods:**

SKY-D is a double-masked, randomised controlled trial funded by the Australian Government’s National Health and Medical Research Council (NHMRC). Participants aged between 16 and 25 years (inclusive) with moderate-to-severe MDD will be randomised to receive either low-dose ketamine (intervention) or midazolam (active control) via subcutaneous injection once per week for 4 weeks. The primary outcome is change in depressive symptoms on the Montgomery-Åsberg Depression Rating Scale (MADRS) after 4 weeks of treatment. Further follow-up assessment will occur at 8 and 26 weeks from treatment commencement to determine whether treatment effects are sustained and to investigate safety outcomes.

**Discussion:**

Results from this trial will be important in determining whether low-dose subcutaneous ketamine is an effective treatment for young people with moderate-to-severe MDD. This will be the largest randomised trial to investigate the effects of ketamine to treat depression in young people.

**Trial registration:**

Australian and New Zealand Clinical Trials Registry ID: ACTRN12619000683134. Registered on May 7, 2019. https://www.anzctr.org.au/Trial/Registration/TrialReview.aspx?id=377513.

## Administrative information

Note: the numbers in curly brackets in this protocol refer to SPIRIT checklist item numbers. The order of the items has been modified to group similar items (see http://www.equator-network.org/reporting-guidelines/spirit-2013-statement-defining-standard-protocol-items-for-clinical-trials/).

**Table:** 

Title {1}	The Study of Ketamine for Youth Depression (SKY-D): study protocol for a randomised controlled trial
Trial registration {2a and 2b}	Australian and New Zealand Clinical Trials Registry ID: ACTRN12619000683134
Protocol version {3}	ANZCTR Registered 07/05/2019
Funding {4}	This trial was funded by the National Health and Medical Research Council and Orygen.
Author details {5a}	Orli S. Schwartz^1−3^, Paul Amminger^2,3^, Bernard T. Baune^1,4,5^, Gillinder Bedi^2,3^, Michael Berk^2,6^, Sue M. Cotton^2,3^, Rothanthi Daglas-Georgiou^2,3^, Nick Glozier ^7–9^, Ben Harrison^1^, Daniel F. Hermens^10^, Emma Jennings^2,3^, Jim Lagopoulos^10,11^, Colleen Loo^12,13^, Sumudu Mallawaarachchi^2,3^, Donel Martin^13,14^, Bethany Phelan^2,3^, Nikki Read^2,3^, Anthony Rodgers^15^, Lianne Schmaal^2,3^, Andrew A. Somogyi^16^, Lily Thurston^2,3^, Amber Weller^2,3,17^, & Christopher G. Davey^1−3^. ^1^ Department of Psychiatry, University of Melbourne, Melbourne, Australia ^2^ Orygen, Melbourne, Australia ^3^ Centre for Youth Mental Health, University of Melbourne, Melbourne, Australia ^4^ Department of Psychiatry, University of Münster, Münster﻿, Germany ^5^ The Florey Institute of Neuroscience and Mental Health, Melbourne, Australia ^6^ Deakin University, IMPACT, The Institute for Mental and Physical Health and Clinical Translation, School of Medicine, Geelong, Australia ^7^ Central Clinical School, Faculty of Medicine and Health, The University of Sydney, Sydney, Australia ^8^ Australian Research Council Centre of Excellence for Children and Families over the Life Course, Sydney, Australia ^9^ Professor Marie Bashir Centre, Royal Prince Alfred Hospital, Sydney, Australia ^10^ Thompson Institute, University of the Sunshine Coast, Sunshine Coast, Australia ^11^ Thompson Brain and Mind Healthcare, Sunshine Coast, Australia ^12^ School of Psychiatry, University of New South Wales, Sydney, Australia ^13^ Black Dog Institute, Sydney, Australia ^14^ School of Clinical Medicine, University of New South Wales, Sydney, Australia ^15^ The George Institute for Global Health, University of New South Wales, Sydney, Australia ^16^ School of Biomedicine, University of Adelaide, Adelaide, Australia ^17^ Victorian Department of Health, Melbourne, Australia
Name and contact information for the trial sponsor {5b}	Trial sponsor: Orygen Contact: Kerryn Pennell Address: 35 Poplar Road, Parkville, Victoria, Australia, 3052 Phone: + 61 3 9966 9100 Email: kerryn.pennell@orygen.org.au.
Role of sponsor {5c}	This is an investigator-initiated trial. The study sponsor and funders had no role in the design of the study, and will not have any role during its execution, analyses, interpretation of the data, or decision to submit results.

## Introduction

### Background and rationale {6a}

Depressive disorders are common mental health conditions and a leading cause of disease burden [1]. In Australia, depression affects 12% of young people aged 15–24 years [2]. Major depressive disorder (MDD) in young people is associated with high rates of relapse [3] and significant developmental disruption that can have effects throughout adult life [4–6].

Existing treatments for young people with depression have significant limitations. Antidepressants have less efficacy in young people than in adults, commonly resulting in only “small and unimportant” reductions in depressive symptoms [7]. The benefits of common medications, particularly selective serotonin reuptake inhibitors (SSRIs), may also not emerge for several weeks [8], and there are concerns that they may increase suicidal thoughts and behaviours in young people [7]. Similarly, psychotherapy has consistently been found to have only modest effects in young people with moderate-to-severe depression [9–11]. There is a compelling need for the development of treatments that can provide rapid and significant alleviation of symptoms in young people with depression. Recent evidence from adult trials (summarised below) indicates that low-dose ketamine holds considerable promise in this regard.

Ketamine, an n-methyl-d-aspartate receptor (NMDAR) antagonist, is a commonly used anaesthetic agent and is on the World Health Organization (WHO) list of essential medicines due to its effectiveness, safety and affordability as an anaesthetic [12]. While ketamine’s antidepressant mechanisms of action are not yet well understood, its antagonism of NMDAR receptors causes downstream effects that are proposed to be relevant, including increased synaptic plasticity and strengthening of excitatory synapses [13].

Since the first randomised control trial (RCT) published over 20 years ago [14], there have been approximately 50 RCTs reporting on the efficacy of ketamine as a treatment for depression and 10 meta-analyses published since 2015. These meta-analyses indicate that racemic ketamine is more effective than placebo or active control in producing treatment response (i.e. reduction in depressive symptoms) and depression remission at 24 h post-administration [15–24], with effects lasting up to 7 days [17–23]. There is also evidence that intravenous ketamine rapidly reduces suicidal ideation in depressed patients, independent of its antidepressant effects [25].

Most published trials have assessed the efficacy of a single dose of racemic ketamine administered intravenously in adults [15], while an emerging evidence base shows that subcutaneous administration has comparable efficacy to the intravenous route [26]. Evidence from open label trials and case studies [18, 19, 22] and a small number of RCTs [27–33] suggests that repeated treatments with racemic ketamine (e.g. twice weekly treatments for between 2 and 4 weeks) lead to prolonged antidepressant effects over the course of treatment.

Only two small studies have examined the efficacy of low-dose ketamine for young people with depression, with both reporting promising results, albeit with significant methodological limitations. First, Cullen and colleagues [34] published a small, open-label study with 13 adolescents (mean age 16.9 years, range 14.5–18.8 years) with depression who were administered 6 intravenous ketamine infusions (0.5 mg/kg for 40 min) over 2 weeks. Five adolescents (39%) showed a clinical response (defined as a 50% reduction in baseline depression symptoms), with 3 of these 5 showing sustained remission at 6-week follow-up.

The second trial, published in 2021 by Dwyer and colleagues [35], was a proof-of-concept RCT with a cross-over design using midazolam as active comparator. The trial included 17 adolescents aged 13–17 years (mean age 15.5 years), and a single dose of ketamine was administered via intravenous infusion at 0.5 mg/kg. Results showed that 13 (77%) adolescents showed a response to ketamine within the first 3 days of treatment, although 5 of these participants also responded to midazolam. The 24-h post-treatment effect size was similar to that observed in midazolam-controlled ketamine trials in adults [36].

In regard to safety, Short and colleagues’ [37] systematic review of ketamine treatment for depression reported ketamine to be generally well-tolerated, with common acute side effects generally not of clinical significance, transient, and spontaneously resolving. Insufficient evidence was available regarding repeated dosing and long-term effects for any conclusions to be drawn about these outcomes. Low-dose ketamine has also been well-tolerated in young people, with a very similar side-effect profile to adults reported [35].

Building on the growing evidence base in adults [33], the Study of Ketamine for Youth Depression (SKY-D) will be the largest RCT to date of low-dose ketamine for young people with moderate-to-severe major depressive disorder (MDD). Key methodological strengths of the trial include a titrated dosing protocol over four treatments to enable investigation of the dose–response relationship, a comprehensive assessment of side-effects, and a 6-month follow-up period to evaluate the longer-term safety profile of this treatment. Midazolam will be used as the active comparator condition on account of its ability to mitigate unmasking given its similar subjective effects and pharmacokinetic and pharmacogenomic profile to ketamine [36]. Finally, the study drugs will be administered via subcutaneous injection, a less-invasive method of administration relative to intravenous infusion that may be more acceptable to young people, and one that is convenient, cost-effective, and readily applicable to clinical implementation [26, 33].

## Objectives {7}

The primary aim of SKY-D is to determine whether a 4-week course of low-dose subcutaneous ketamine is an effective adjunct to treatment-as-usual, relative to an active control condition, in young people (16 to 25 years of age) with moderate-to-severe MDD.

The secondary aims are to determine whether the intervention reduces suicidality and improves rates of depression remission, anxiety symptoms, and quality of life, relative to an active control condition.

Exploratory aims include a biomarker/mechanistic study and assessment of safety. Specifically, SKY-D will collect neuroimaging data, blood markers, sleep variables, and acute drug effects to conduct an exploratory study of ketamine’s effects, with examination of its mechanisms of action and identification of predictors of response. SKY-D will also assess the safety of low-dose subcutaneous ketamine in this age group, including assessment of adverse effects, psychotomimetic and dissociative symptoms, cognitive function, clinical blood markers, and abuse liability.

It is hypothesised that a 4-week course of low-dose ketamine, compared to a therapeutically inactive control treatment (midazolam), will reduce depression symptoms in young people with MDD. It is also hypothesised that ketamine treatment will increase the proportion of participants who achieve remission of their MDD, as well as reduce suicidal thoughts and behaviours, reduce anxiety symptoms, and improve quality of life relative to control, and that it will be a safe and tolerable treatment.

## Trial design {8}

SKY-D is a randomised, controlled, double-masked, parallel-group, multicentre superiority trial. The study will enrol 140 participants aged 16–25 years with MDD. Participants will be randomised 1:1 into two study arms, receiving either low-dose subcutaneous ketamine or a control treatment that is therapeutically inactive (midazolam), given once a week for 4 weeks.

The primary endpoint is at week 4, 7 days after completion of the treatment phase, with further assessment at weeks 8 and 26 to assess safety and sustained treatment effects.

## Methods: participants, interventions, and outcomes

### Study setting {9}

The trial will be conducted at three sites in Australia: at the Royal Melbourne Hospital (Victoria), the Royal Prince Alfred Hospital in Sydney (New South Wales), and the Thompson Institute, University of the Sunshine Coast (Queensland).

### Eligibility criteria {10}

Participants will be included if they meet all the following criteria:

1. Aged 16–25 years inclusive at the time of providing informed consent.
2. Current MDD as assessed using the Structured Clinical Interview for DSM-5 (SCID-5 [38]).
3. Montgomery-Åsberg Depression Rating Scale (MADRS) ≥ 22—equivalent to moderate-to-severe depression—within 7 days of the first treatment visit.
4. Treatment with either a stable dose of an antidepressant or no antidepressant medication for ≥ 2 weeks.
5. Ability to provide written informed consent (including both adequate intellectual capacity and fluency in the English language).

Participants will be excluded if they meet any of the following criteria:

1. Severe disturbance such that the young person would be unable to comply with the requirements of informed consent or comply with the study protocol, as determined by the trial doctor.
2. History of psychosis or bipolar disorder (assessed with SCID-5).
3. Any unstable medical condition or contraindication to ketamine or midazolam use (as indicated in the Product Information forms for ketamine and midazolam).
4. Any history of a ketamine use disorder of any severity or presence of a substance use disorder of at least moderate severity (according to DSM-5 criteria, [39]) within the preceding 6 months, as determined by the trial doctor.
5. People of child-bearing potential who are pregnant or currently breastfeeding or who are at risk of pregnancy because they are not using effective contraception.
6. Participation in any other clinical intervention trial from SKY-D baseline to the week 8 follow-up.

### Who will take informed consent? {26a}

Written informed consent will be obtained from all participants before they can enter the study.

All eligible participants will have the study explained by the principal investigator (PI) or trained delegate and will be provided with written information. They will receive a full explanation of the aims of the study, the risks, and benefits of taking part and will be provided with ample opportunity to ask questions.

For participants who are aged 16 or 17 years, two requirements for obtaining consent must be met: the participant must consent on their own behalf, and all possible attempts must be made to obtain additional written, informed consent from a parent or legal guardian. When it is not possible to obtain consent from a parent/guardian, consent may be obtained from the young person alone if they have been assessed by a trial doctor or delegate to have the capacity to provide informed consent.

### Additional consent provisions for collection and use of participant data and biological specimens {26b}

No ancillary studies will be conducted.

## Interventions

### Intervention description {11a}

Participants will be randomised to receive either low-dose ketamine (treatment group) or midazolam (active control group). Treatment will be provided once a week over 4 weeks. Participants will be assessed and closely monitored by trained trial staff for 4 h following the first treatment and for 2 h for the remaining three treatments.

All participants will receive a starting dose (level 1) of 0.6 mg/kg of ketamine or 0.03 mg/kg of midazolam at the first treatment. The MADRS will be administered on arrival prior to treatments 2, 3, and 4 (i.e. 7 days after the previous treatment). Participants with inadequate treatment response (defined as less than a 50% reduction relative to baseline scores on the MADRS) will be provided with an increased dose. The dosage will increase stepwise, with an increase at the first step to 0.75 mg/kg ketamine or 0.0375 mg/kg midazolam (level 2), and at the second step to 0.9 mg/kg of ketamine or 0.045 mg/kg of midazolam (level 3, the maximum dose). Those who are unable to tolerate the starting dose (level 1) will have their dosage reduced for subsequent treatments to a minimum of 0.5 mg/kg of ketamine and 0.025 mg/kg of midazolam (level 0). If a participant is unable to tolerate dose levels 2 or 3, they will have their dosage reduced to the previously tolerated level. Determination of tolerance is based on the participant’s subjective report and clinician judgement.

Treatments will be administered by a trained nurse or doctor via subcutaneous injection into the participant’s abdomen. The injection site will be rotated (i.e. from left to right side at each treatment) to avoid repeated injections in the same area.

### Explanation for the choice of comparators {6b}

Midazolam will be used as an active control treatment following its use as a comparator in several recent ketamine trials [33, 35, 40]. Midazolam causes subjective effects that mitigates participant unmasking to treatment allocation but does not have any known antidepressant effects [36].

### Criteria for discontinuing or modifying allocated interventions {11b}

A participant may be discontinued from the treatment voluntarily or by the study team for reasons including, but not limited to:

1. The site PI, in consultation with the treating doctor, believing that for safety reasons it is in the best interest of the participant to be discontinued because participation is interfering with clinical risk management
2. The participant developing any unstable medical condition, or contraindication to ketamine or midazolam, or beginning a pharmaceutical treatment that is contraindicated for ketamine or midazolam use
3. The participant beginning recreational ketamine or midazolam use or developing a substance use disorder of at least moderate severity (SCID-5)
4. The participant becoming pregnant
5. The participant being unwilling or unable to comply with required study procedures

Discontinuing or modifying treatments will have no implications for scheduled follow-up assessments. A participant will be withdrawn from follow-up only following the revocation of consent or when the site PI, in consultation with the treating doctor, believes that it is in the best interest of the participant to be withdrawn.

### Strategies to improve adherence to interventions {11c}

A participant’s treatment dosage will only be titrated up if the participant has indicated that they could tolerate an increased dose, while also considering the observations of the clinical staff managing the treatments.

Participants will be provided with appointment confirmation details prior to each trial visit, and transport may be provided upon request.

### Relevant concomitant care permitted or prohibited during the trial {11d}

This trial is examining ketamine as an adjunct to treatment-as-usual. As such, all medications that are being used to treat the participant’s depression are permitted. Treating doctors will be asked that their patients maintain a stable dose of medication through the trial period; any deviations from this will be recorded.

No medications are specifically prohibited. If any medication is taken, the medication name, dose, route, frequency of dosing, and reason for use will be recorded.

### Provisions for post-trial care {30}

Treating doctors who refer participants will be notified when treatment commences and concludes and will be advised of the participant’s progress and any significant side effects that require follow-up.

### Outcomes {12}

#### Primary outcome measure

The primary outcome is the difference between the MADRS score at week 4 (7 days following the fourth and final treatment) compared with the baseline MADRS score. The MADRS, an observer-rated depression scale, is used widely in depression treatment trials because it is both efficient to administer and psychometrically sound [41, 42].

#### Secondary outcome measures

Secondary outcome measures are defined as follows:

- Change in the MADRS at week 8 compared with baseline MADRS
- Change in the MADRS at 24-h after each of the four treatment sessions, compared with the pre-treatment MADRS recorded before each treatment (the baseline MADRS is considered the pre-treatment MADRS for treatment 1)
- Change in self-rated depression symptoms between baseline and weeks 4 and 8 using the Quick Inventory of Depression Symptomatology – Self Report version (QIDS, [43])
- Remission of depression, defined as MADRS score ≤ 10, at weeks 4 and 8
- Change in suicidal ideation between baseline and weeks 4 and 8 using the self-rated Suicidal Ideation Screen (SIS, [44])
- Absence of suicidal thoughts at weeks 4 and 8, defined as a MADRS Suicidality Item score of 0
- Absence of a suicide attempt at weeks 4 and 8, defined as a score of 0 on the Columbia-Suicide Severity Rating Scale (C-SSRS, [45]) ‘actual attempt’ criterion
- Researcher-rated response to treatment at weeks 4 and 8, defined as a Clinical Global Impression – Improvement (CGI-I, [46]) score of ≤ 2 (‘much’ or ‘very much’ improved)
- Participant-rated response to treatment at week 4, defined as a Patient Global Impression – Improvement (PGI-I, [46]) score of ≤ 2 (‘much’ or ‘very much’ improved).
- Change in anxiety scores from baseline to weeks 4 and 8 using the self-report Generalized Anxiety Disorder 7-item (GAD-7, [47]) scale
- Change in quality of life from baseline to weeks 4 and 8 using the self-report Assessment of Quality of Life – 8 Dimensions (AQoL-8D, [48])
- Change in social and occupational functioning from baseline to weeks 4 and 8 using the researcher-rated Social and Occupational Assessment Scale (SOFAS, [49])

#### Exploratory outcome measures

Several biological and functional markers of ketamine’s effect will be investigated pre- and post-treatment to better understand its mechanisms of action and identify predictors of response:

- Magnetic resonance spectroscopy will be conducted to examine brain spectra of glutamate, glutamine, and glutathione
- Functional magnetic resonance imaging (fMRI) tasks (resting state and task-related fMRI, including a self-referential processing task and emotional face processing task) will be analysed to identify baseline predictors of treatment response and brain correlates of response
- Blood samples will be used to conduct pharmacokinetic analysis of plasma concentrations of ketamine and its major active metabolites and analyse biomarkers related to treatment response (e.g. brain derived neurotrophic factor), including genetic predictors of this variability
- Objective (actigraphy) and subjective measures of sleep, including the Pittsburgh Sleep Quality Index (PSQI, [50]) and the revised Morningness-Eveningness Questionnaire (rMEQ, [51]) will be analysed to assess whether sleep change is a mechanism underlying ketamine’s rapid anti-depressant effects and whether sleep effects predict treatment response to ketamine
- Acute drug effects will be assessed using the Hood Mysticism Scale (HMS, [52]) and the Drug Effects Questionnaire (DEQ, [53]) to investigate whether such effects predict treatment response
- Finally, the early response to ketamine (measured by the MADRS at 24 h after each treatment) will be investigated as a potential predictor of sustained anti-depressant response (measured by the MADRS at weeks 4, 8, and 26)

The safety of low-dose subcutaneous ketamine will be assessed by analysing changes in:

- Cognitive function assessed using a computerised battery via Cogstate
- Clinical blood parameters
- Adverse events assessed using the Ketamine Side Effects Tool (KSET, [54])
- Psychotomimetic effects using subscales from the Brief Psychiatric Rating Scale (BPRS, [55])
- Dissociative effects using the Clinician Administered Dissociative Symptoms Scale (CADSS, [56])
- And substance use and related problems using the Alcohol, Smoking and Substance Involvement Screening Test (ASSIST, [57]) and modified Brief Substance Craving Scale (BSCS, [58])

### Participant timeline {13}

The SKY-D timeline of enrolment, intervention, and assessment is presented in Table 1.

**Table 1:** Schedule of assessments and trial visits

**Time point**	Baseline	Treatment 1	24-h follow-up	Treatment 2	24-h follow-up	Treatment 3	24-h follow-up	Treatment 4	24-h follow-up	Week 4 follow up	Week 8 follow-up	Week 26 follow-up
**Day**	− 21 to − 1	0	1	7	8	14	15	21	22	28^a^	56^b^	182^c^
Informed consent	X											
Medical and psychiatric review	X											
Demographics	X											
Physical examination (vital signs)^d^	X	X		X		X		X		X		
Concomitant pharmaceutical treatment	X	X		X		X		X		X	X	X
Medical condition	X	X		X		X		X		X	X	X
Doctor’s provisional eligibility checklist^e^	X											
Final eligibility checklist^f^	X											
**Diagnosis**												
SCID-5 (modules A, B/C Screen, and D)	X											
**Randomisation**^g^	X											
**Intervention administered**^h^ *ketamine vs midazolam*		**X**		**X**		**X**		**X**				
Clinician prediction of treatment response	X											
Masking assessment										X		
**Biological measures**												
Pregnancy (serum)^i^	X											
Clinical bloods	X									X		
Research bloods^j^	X	X^k^								X		
**Symptomatology**												
Montgomery-Asberg Depression Rating Scale (MADRS)^a^	X^a^		X^a^	X^a^	X	X	X	X	X	X	X	X
Quick Inventory of Depression Symptomatology (QIDS)	X			X		X		X		X	X	
Clinical Global Impression-Severity Scale (CGI-S)	X			X		X		X		X	X	
Clinical Global Impression-Improvement Scale (CGI-I)				X		X		X		X	X	
Patient Global Impression-Improvement Scale (PGI-I)				X		X		X		X		
Generalized Anxiety Disorder 7-item Scale (GAD-7)	X			X		X		X		X	X	
**Suicidality**												
Suicidal Ideation Screen (SIS)	X			X		X		X		X	X	
Columbia Suicide Severity Rating Scale (CSSRS)	X			X		X		X		X	X	
**Substance use**												
Brief Substance Craving Scale (BSCS)	X			X		X		X		X	X	X
Alcohol, Smoking and Substance Involvement Screening Test (ASSIST)	X									X	X	X
**Functioning/quality of life**												
Assessment of Quality of Life (AQoL-8D)	X									X	X	
Social and Occupational Functioning Assessment Scale (SOFAS)	X									X	X	
**Sleep**												
Actigraphy^l^	X	X	X	X				X	X	X		
Consensus Sleep Diary (CSD)^m^	X	X	X	X				X	X	X		
Pittsburgh Sleep Quality Inventory (PSQI)	X									X	X	
Revised Morningness-Eveningness Questionnaire (rMEQ)	X											
**Cognitive assessment**												
Cogstate	X									X		
**Brain scan**												
Magnetic resonance imaging (MRI)^n^		X^o^								X		
**Drug and adverse effects**												
Adverse events	X	X	X	X	X	X	X	X	X	X	X	X
Ketamine Side-Effects Tool (KSET)^p^	X	X		X		X		X		X	X	X
Clinician Administered Dissociative Symptoms Scale (CADSS)^q^	X	X		X		X		X				
Brief Psychiatric Rating Scale (BPRS)^r^	X	X		X		X		X				
Hood Mysticism Scale (HMS)^s^		X										
Drug Effects Questionnaire (DEQ)^t^		X		X		X		X				

^a^The week 4/day 28 follow-up assessment will be completed within a window of ± 3 days of day 28. It will ideally be conducted face-to-face at the research site but may be conducted via a home visit or via telephone/telehealth video call if necessary

^b^The week 8/day 56 follow-up assessment will be completed within a window of ± 3 days of day 56. It will be conducted via telephone/telehealth video call

^c^The week 26/day 182 follow-up assessment will be completed within a window of ± 7 days of day 182. It will be conducted via telephone/telehealth video call

^d^During treatment visits, vital signs will be assessed prior to treatment and four times after treatment (at 15, 30, 60, and 120 min). After the first treatment, vital signs will also be assessed at 240 min after treatment

^e^To be completed by a trial doctor after the Medical and Psychiatric Review on entry to the study

^f^To be completed once all data pertaining to eligibility has been finalised. Must not be completed earlier than day − 7

^g^Randomisation can occur after the completion of the final eligibility checklist (which is to be finalised no earlier than day − 7) and up to and including day − 1

^h^Treatment can be administered within ± 3 days of days 7, 14, and 21, respectively, with a minimum of 4 days between treatments. Missed sessions outside this window cannot be made up

^i^Pregnancy will be tested using serum at baseline. Participants will be re-tested during the treatment phase only if they indicate that there is a possibility that they may be pregnant

^j^Research blood samples are optional

^k^Research blood collected 4 h after first treatment

^l^Actigraphy is optional. For consenting participants, actigraphy will be collected for approximately 10–14 days, starting from approximately 3–7 days before the first treatment, until the second treatment visit (i.e. day 7). Actigraphy will be collected again over the 7 nights between the final treatment (day 21) and the week 4 follow-up (day 28)

^m^The Consensus Sleep Diary will be recorded for each night that actigraphy is collected, and only for those who consent to participate in the actigraphy assessment

^n^The MRI scans are optional

^o^The first MRI scan may be conducted during the baseline phase, or on the first treatment visit prior to the administration of medication

^p^KSET screener and baseline administered at baseline; KSET acute treatment administered before and at 60 and 120 min after each treatment (plus at 240 min after the first treatment); KSET follow-up administered at weeks 4, 8, and 26 follow-up

^q^Administered at baseline and at 60 and 120 min after each treatment

^r^Administered at baseline and at 60 and 120 min after each treatment

^s^Administered 120 min after the first treatment

^t^Administered 60 min after each treatment

### Sample size {14}

The target sample size for the study is 140 participants.

The study is powered at 80% to detect a pre-post treatment standardised mean difference on the MADRS for ketamine versus midazolam of 0.5 at *p* = 0.05 and allows for a 10% attrition rate. This calculation uses an effect size estimate based on recent meta-analysis for change in depressive symptoms 7 days after a single dose of ketamine [21]. The primary analyses will be based on the intention-to-treat inclusion of all participants randomised in the study with baseline data.

### Recruitment {15}

Study staff at each site will communicate with treating doctors within their networks who will be asked to identify potentially suitable patients (i.e. young people with depression who meet the eligibility criteria). Information about the study and the specific eligibility criteria will be provided to treating doctors. Advertising/media releases may be conducted to raise awareness about the study. In addition, the study will be listed on the websites of organisations associated with the trial. Young people will not be enrolled in the trial without a referral from their treating doctor.

## Assignment of interventions: allocation

### Sequence generation {16a}

The randomisation sequence will be generated by a statistician independent of the study. Participants will be randomised to receive ketamine or the control treatment (midazolam) at a ratio of 1:1, stratified by trial site (Melbourne, Sydney and Sunshine Coast) and age (under 21 years and 21 years and over). Random permutated blocks will be used.

### Concealment mechanism {16b}

The randomisation sequence will be programmed into Orygen’s online Research Project Management System (RPMS) which ensures that the allocation sequence remains concealed.

### Implementation {16c}

Following completion of the baseline assessment and eligibility checklists in the electronic case record form (eCRF), research staff will identify participants ready for randomisation. The project manager will confirm all eligibility criteria have been met and will request randomisation via the RPMS. Relevant study staff will be notified of the 4-digit randomisation ID through an automatically generated email from the RPMS.

## Assignment of interventions: masking

### Who will be masked {17a}

The participants, site research and clinical staff, sponsor (Orygen), monitor, and statistician will all be masked with respect to treatment allocation. Treatment allocation will not be revealed until all data collection for the study is completed. Only the pharmacists, unmasked monitor, and unmasked statistician will have access to group allocation. Treatment allocation may be revealed to the data safety and monitoring board (DSMB) if required, in accordance the DSMB Charter for this trial.

### Procedure for unmasking if needed {17b}

There will be 24-h emergency access to treatment allocation for the PI or qualified delegate in the case that the randomisation code for an individual participant needs to be broken. Individual participant randomisation codes will only be broken in the case of emergency, where the PI feels a participant cannot be adequately treated without knowing the identity of the treatment administered. The unmasking procedure is conducted through RPMS, and the treatment allocation is only seen by the qualified team member who conducts the unmasking.

All details of the code-break will be documented including the reason the code was broken, and which personnel have been unmasked.

If the code is prematurely broken for a participant, the participant will be discontinued from the study intervention. However, if, in the considered opinion of the PI or qualified delegate, it is safe and appropriate for a participant to continue with study assessments after unmasking, the participant may do so.

## Data collection and management

### Plans for assessment and collection of outcomes {18a}

Assessments will be conducted in-person or via videocall or telephone. Self-report assessments will be completed either in-person or online.

Study staff administering the primary outcome measure (the MADRS) will be required to achieve adequate inter-rater reliability relative to a ‘gold standard’ set of MADRS ratings determined by the sponsor prior to beginning to administer assessments on the trial. Adequate inter-rater reliability is defined as an intra-class correlation of ≥ 0.8 with the ‘gold standard’ ratings.

### Study assessment procedures

Study assessments will be conducted over a 29-week period, with 12 assessment time points: baseline (up to a 3-week period), the treatment period (which includes 8 assessments spanning week 0 to week 3), and follow-ups at weeks 4, 8, and 26, as follows:

- 1. Baseline (day − 21 to − 1).

While the baseline period can range from day **− **21 to day **− **1, participants must record a MADRS score of ≥ 22 within 7 days prior to day 0. The baseline assessment includes screening for eligibility and baseline assessment of all the outcome measures prior to randomisation (see Table 1).

- 2. Treatment phase (days 0, 1, 7, 8, 14, 15, 21, and 22).

Participants will attend the hospital/research facility for treatments 1–4 on days 0, 7, 14, and 21, respectively. Participants will be closely monitored, with vital signs recorded immediately before treatment and 15, 30, 60, 120, and (for treatment 1 only) 240 min after treatment. Adverse and psychotomimetic effects will be assessed before and 60 and 120 min after treatment. Participants will complete a battery of psychosocial assessments prior to dosing administration at treatments 2, 3, and 4 (see Table 1).

Participants will be followed up by telephone 24 h after each treatment to determine their early treatment response using the MADRS and review for adverse effects.

- 3. Primary endpoint; week 4 follow-up (day 28).

Participants will return to the hospital/research facility for the primary endpoint assessment, including the battery of all outcome measures outlined in Table 1.

- 4. Week 8 (day 56) and week 26 (day 182) follow-ups.

Participants will be contacted by telephone/video call to complete a brief battery of psychosocial assessments and adverse effects outlined in Table 1.

### COVID-19 risk mitigation procedures

All sites must comply with local requirements in regard to COVID-19 restrictions and safety procedures.

To reduce the requirement for in-person contacts, some components of the SKY-D protocol may be conducted remotely via telephone or video call when required. Components of the protocol that must be conducted in-person include treatment administration and post-treatment monitoring, the collection of blood samples, cognitive assessment, physical examinations, and MRI scans.

### Study measurements procedures

- 1. Participant psychosocial self-reports.

A battery of self-report measures assessing a range of functioning domains including depression and anxiety symptoms, suicidality, substance use, sleep, and quality of life will be administered at various time points as detailed in Table 1.

- QIDS: a 16-item self-report instrument assessing the severity of the nine diagnostic symptom criteria of MDD used in the DSM over the past 7 days
- PGI-I: a 7-point scale that requires the patient to assess how much their illness has improved or worsened relative to the baseline state before the intervention
- SIS: a 3-item item questionnaire assessing the frequency, intensity and controllability of suicidal ideation
- GAD-7: a 7-item questionnaire assessing symptoms of generalised anxiety over the past 2 weeks
- BSCS: a 4-item, self-report instrument assessing drug craving over a 24-h period. Intensity and frequency of craving are recorded on a five-point Likert scale. Questions will be modified to specifically address use and craving of ketamine
- AQoL-8D: a 35-item questionnaire assessing eight domains of QoL, including independent living, happiness, mental health, coping, relationships, self-worth, pain, and senses
- PSQI: a 9-item self-report inventory designed to assess self-reported sleep quality and disturbances and the impact of poor sleep on daytime functioning
- rMEQ: 5 items which determine individual chronotype on a single scale, where higher scores indicate a tendency towards morningness

- 2. Psychosocial rating scales.

A battery of researcher-rated scales assessing a range of functioning domains including depression symptoms, suicidality, substance use, and socio-occupational functioning will be administered at multiple time points as detailed in Table 1.

- MADRS: a 10-item scale that evaluates core symptoms of depression. Nine items are based on patient report, and one is based on clinician observation
- CSSRS (baseline and follow-up): patients are asked a series of questions about suicidal thoughts and behaviours. The number and choice of questions asked depend on the patient’s answers
- CGI-S/I: a 7-point scale that requires the researcher to assess the severity of the illness at baseline and how much the patient’s illness has improved or worsened relative to baseline
- SOFAS: a 100-point scale assessing an individual's level of social and occupational functioning over a specified time period
- ASSIST: screens for all levels of problem or risky substance use. It consists of eight questions covering use of tobacco, alcohol, cannabis, cocaine, amphetamine-type stimulants (including ecstasy) inhalants, sedatives, hallucinogens, opioids and ‘other drugs’

- 3. Drug and adverse effects.

Side-effects and psychotomimetic effects will be assessed at baseline and at each treatment, and acute drug effects will be assessed during treatments.

- BPRS: a subset of five items assessing psychotic symptoms (grandiosity, suspiciousness, hallucinations, unusual thought content, and conceptual disorganisation). It is administered at baseline and at 60 and 120 min after each of the 4 treatments
- CADSS: 27 items assessing dissociative symptoms including 19 items based on patient report and 8 based on researcher observation. It is administered at baseline and at 60 and 120 min after each of the 4 treatments
- KSET: a scale specifically designed to evaluate adverse effects of low-dose ketamine treatment. The scale has been modified for SKY-D to include assessment of screening for contraindications and adverse effects of midazolam. There are four modules of the KSET: Screening, Baseline, Acute Treatment, and Follow-up. The modules assess contraindications for ketamine (and midazolam) treatment and vital signs and adverse events before, during, and after treatments
- Adverse effects will be recorded at each participant assessment
- HMS: 9 items assessing mystical experiences during acute drug effects. It is administered during the first treatment, 120 min after treatment
- DEQ: a 5-item scale assessing acute drug effects. It is administered at 60 after each of the 4 treatments

- 4. Cognitive function.

Cognitive functioning will be assessed using the computerised Cogstate instrument at baseline and at week 4 follow-up. The following seven subtests will be administered: International Shopping List Task (assesses verbal learning and memory), Detection (simple reaction time), Identification (choice reaction time), One Card Learning (visual memory), 2-Back (working memory), Set-Shifting Task (set-shifting/executive function), and International Shopping List Task (delayed recall).

- 5. Clinical laboratory tests.

The following clinical blood tests will be run at baseline and week 4 follow-up: full blood examination; liver function test; thyroid function test; and electrolytes, urea, and creatinine test. As appropriate, participants of child-bearing potential will also be tested for human chorionic gonadotropin at baseline.

- 6. Research blood tests.

Optional blood samples to assess for treatment biomarkers will be collected at baseline, 4 h after the first treatment administration, and at the week 4 follow-up assessment. Paxgene tubes will be used to collect RNA, and serum and plasma will be collected for protein analyses. Plasma concentrations of ketamine and its major active metabolites, as well as treatment response biomarkers such as brain derived neurotrophic factors and genetic markers, will be analysed. In relation to genetic analyses, single-nucleotide polymorphisms and copy number variants for genes that have been associated with MDD, suicidal ideation, antidepressant treatment response and adverse effects, and the glutamatergic system will be examined.

- 7. MRI scans.

Participants at the Melbourne and Sunshine Coast sites (*n* = 105) will undergo optional MRI scanning on identical 3T Siemens Prisma scanners, with acquisition of spectroscopic and functional images at baseline and week 4 follow-up. In vivo brain spectra of glutamate, glutamine, and glutathione will be recorded from a 2 × 2 × 2 cm voxel placed in the anterior cingulate cortex using an optimised PRESS sequence [59] and from a 2 × 2 × 2 cm voxel placed in the posterior cingulate cortex using a MEGAPRESS sequence [60]. Participants will also complete the following: (i) a structural scan; (ii) resting-state fMRI (analysed using our previously developed methods [61–63]; (iii) a self-appraisal task we have developed to specifically activate the medial prefrontal cortex [64]; and (iv) a basic emotional faces task, similar to those used in previous ketamine imaging studies [65, 66]. For both the spectroscopic and fMRI analyses, we will examine baseline predictors of treatment response, and brain correlates of response (in both cases examining factors that pertain to treatment response overall, and to ketamine response specifically).

- 8. Actigraphy.

Wrist-worn actigraphy devices (GENEActiv) will be worn (optional) at two time-points: from baseline to the second treatment visit (from approximately days −7 to 7) and from the final treatment visit until the week 4 follow-up (days 21 to 28). Participants will be asked to complete the Consensus Sleep Diary (CSD) [67] each night they wear the actigraphy device. The CSD is a 9-item questionnaire that prompts participants to record bedtime, sleep onset latency, number of nocturnal awakenings, wake time, rise time, and perceived quality of sleep.

- 9. Diagnostic interview.

At baseline, participants will be interviewed using the SCID-5 (modules A, B/C Screen, and D, assessing mood disorders and psychotic symptoms to determine eligibility), a semi-structured clinical interview to assess current and lifetime history of psychopathology.

- 10. Clinician prediction of treatment response.

At baseline, the study doctor will complete a two-item questionnaire predicting treatment response in relation to depression symptoms, on the assumption that the participant receives ketamine treatment.

- 11. Masking assessment.

At the week 4 follow-up assessment, participants and researchers will be asked three questions to assess the effectiveness of masking to treatment allocation.

### Plans to promote participant retention and complete follow-up {18b}

Every reasonable effort will be made to retain all randomised participants until the final week 26 follow-up. Participants are advised of the visit schedule on enrolment and are provided with a one-page flow-chart of study visits. In addition, participants will receive appointment reminders prior to each visit, as well as a ‘thank you’ letter after the week 8 follow-up that reminds them about the final week 26 follow-up. Research staff will collect the names and contact details of up to two contacts for each participant (e.g. parents, friends, partners), in addition to the referring doctor, in case research staff are unable to contact the participant during the trial.

### Data management {19}

An eCRF within Orygen’s RPMS will be used for this study. Data that is entered directly into the eCRF will be transmitted via a secure website. Source documentation that substantiates the information collected in the eCRF will be collected in either electronic or paper form and may be maintained indefinitely (or for a minimum of 15 years) at the site from completion of the study (database lock). Electronic source documents will be stored on a secure server. In instances of direct data entry, the eCRF will be considered as source.

### Confidentiality {27}

The maintenance of confidentiality will be in accordance with Australian national data and privacy legislation. All participant data and biological samples will be identified by a unique participant identification number and their initials. Participants’ study information will not be released outside of the study without the written permission of the participant, except as necessary for data monitoring purposes, or by authorised representatives of Orygen, regulatory authorities, Human Research Ethics Committees (HRECs), or for safety or mandatory reporting purposes. Study data will be safely stored in restricted access, password-protected, secure computer databases as well as in paper form in locked filing cabinets.

### Plans for collection, laboratory evaluation, and storage of biological specimens for genetic or molecular analysis in this trial/future use {33}

Research blood samples of consenting participants will be stored at each of the sites (or an approved peripheral storage facility) for the duration of the trial, after which they will be transferred to an Australian biobank. The biobank will comply with the National Statement on Ethical Conduct in Human Research (National Health and Medical Research Council, 2007 [Updated 2018]), and the transfer of the samples will only occur following review and approval by a recognised HREC.

## Statistical methods

### Statistical methods for primary and secondary outcomes {20a}

The primary analyses will be based on the intention-to-treat (ITT) inclusion of all participants randomised and will compare the intervention group (ketamine) with the active comparator group (midazolam). Wherever applicable, we will use generalised linear mixed models to analyse both efficacy and safety outcomes, as these models have a number of features relevant to this study: (i) tolerance of missing data; (ii) allowance of both normally distributed outcomes (e.g. MADRS scores) and non-normal outcomes (e.g. binary measures such as remission) in the models; and (iii) incorporation of random effects to allow for inter-individual differences, especially for longitudinal data and site effects [68].

Change analyses for the secondary outcomes will also use generalised linear mixed models. Exploratory outcomes will be analysed using correlational and regression models to examine associations between bio- and functional markers and clinical outcomes. More sophisticated mediational models may also be used to investigate mechanisms of action.

For safety data, the following outcomes will be examined by treatment group: all adverse effects, treatment-related adverse effects, serious adverse effects, and adverse effects leading to permanent discontinuation of the study drug.

### Interim analyses {21b}

An unmasked statistician will prepare summary data pertaining to the primary outcome variable (MADRS score) once 40 randomised participants have completed the primary endpoint (week 4 follow-up) for review by the data safety and monitoring board (DSMB) at a closed meeting. Data will be presented for ‘Arm A’ and ‘Arm B’ to maintain masking of the DSMB. The following data will be reviewed:

- MADRS data (primary outcome) from baseline to the week 4 follow-up;
- Dose level received by each participant at each treatment;
- Number of treatments received by each participant; and
- Safety data.

On the basis of this review, the DSMB will make a recommendation as to whether the trial should be continued, modified, or discontinued.

### Methods for additional analyses (e.g. subgroup analyses) {20b}

Nothing planned.

### Methods in analysis to handle protocol non-adherence and any statistical methods to handle missing data {20c}

All randomised participants will be included in the primary analyses based on an intention-to-treat approach, using generalised linear mixed models that tolerate missing data.

For missing data, all approaches involve assumptions that are unverifiable, and the priority will be to minimise missing data [69]; accordingly, this study has a protocol for follow-up that maximises data in all participants, whether they continue or discontinue treatment, as described previously. Second, for each outcome, the most plausible analysis, given the nature of the missing data, will form the main analysis but will be supplemented with sensitivity analyses examining other approaches.

### Plans to give access to the full protocol, participant-level data, and statistical code {31c}

SKY-D’s data sharing statement is available on the Australia and New Zealand Clinical Trials Registry (ACTRN12619000683134). Briefly, deidentified individual participant data collected during the trial will be shared immediately following publication and for a further 3 years to investigators whose proposed use of the data has been approved by an independent review committee. The study protocol, statistical analysis plan, and informed consent form will also be shared upon request.

## Oversight and monitoring

### Composition of the coordinating centre and trial steering committee {5d}

The Melbourne site, led by the co-ordinating principal investigator (CPI), is the co-ordinating centre for this trial and will offer operational assistance to all sites. The CPI is supported by the project manager, sponsor representatives, study statistician, database manager, and trial steering committee. The trial steering committee comprises all PIs and study investigators and will initially meet monthly and then quarterly once the trial is underway. An operations group comprising the PIs and key personnel from each site will meet monthly. Individual site teams will meet weekly.

### Composition of the data monitoring committee, its role and reporting structure {21a}

A DSMB has been established for SKY-D. All members of the DSMB are independent of the study investigators and the sponsor, Orygen. The primary responsibilities of the DSMB are to:

1. Periodically review and evaluate the accumulated clinical trial data for participant safety and clinical trial conduct progress, and;
2. Make recommendations to Orygen concerning the continuation, modification, or termination of the SKY-D Trial on an ongoing basis. The DSMB considers clinical trial-specific data as well as relevant background knowledge about the disease, investigational product/intervention, or population under trial.

### Adverse event reporting and harms {22}

Adverse events (AEs) are defined as any development of a side-effect or untoward medical condition, or the deterioration of a pre-existing medical condition, following or during exposure to a study intervention, whether or not considered causally related to the study intervention. For the purposes of safety reporting, any research activity will be considered part of the ‘study intervention’.

An AE can therefore be any unfavourable and unintended clinical sign (including an abnormal laboratory finding), symptom, observation, or disease temporally associated with the use of an intervention, whether or not related to the intervention.

Serious adverse events (SAEs) are defined as any untoward medical occurrence that at any administration level—results in death; is life-threatening; requires inpatient hospitalisation or prolongation of existing hospitalisation; results in persistent or significant disability/incapacity; is a congenital anomaly/birth defect; or is an important medical event that although not immediately life-threatening or resulting in death or hospitalisation, based upon appropriate medical and scientific judgment—may jeopardise the participant and/or require intervention to prevent one of the outcomes listed above.

All AEs and SAEs will be recorded in the source documentation and assessed by trial doctor as to causality (related, possibly related, or not related to the study intervention) and severity (mild, moderate or severe).

All SAEs will be reported to site sponsor by the site principal investigator within 24 h of the reporter being aware of event and to the HREC in accordance with HREC requirements.

AEs will be followed up until the AE resolves, stabilises, or the participant is lost to follow-up.

### Frequency and plans for auditing trial conduct {23}

Study monitoring will be performed in accordance with applicable regulations, International Conference for Harmonisation – Good Clinical Practice (ICH-GCP), and Orygen Standard Operating Procedures. The Orygen monitor will regularly contact and visit the sites to monitor study progress, confirm protocol, regulatory and ethical adherence, confirm data accuracy, and provide information and support.

Masked monitoring at each site will be conducted as follows:

- The initial monitoring visit will occur 2–4 weeks after ≥ 2 participants complete the baseline or partial baseline assessment.
- Subsequent visits will occur within 3 weeks following the baseline visit for every 10th participant, with at least two visits per year.
- The close out visit will occur within 3 months of the last participant’s last.

Unmasked monitoring at each site will be conducted whenever ≥ 10 participants have completed the intervention period (or less frequently if the first unmasked monitoring visit is satisfactory). The close-out visit will be conducted once the intervention period has been completed by the last participant at each site.

### Plans for communicating important protocol amendments to relevant parties (e.g. trial participants, ethical committees) {25}

Study procedures will not be changed without the mutual agreement of the CPI and the sponsor, Orygen, and will be communicated to all investigators and relevant site personnel.

Protocol amendments must be approved by the HREC before implementation unless the safety of participants is at risk.

If an amendment substantially alters the study design or increases the potential risk to participants, then re-consenting of currently enrolled participants may be required, as determined by the HREC.

### Dissemination plans {31a}

After approval by the CPI, all investigators, and the trial statistician, the results of the study will be published in peer-reviewed scientific journals and presented at scientific conferences. Where participants have asked to see the results of the study, these results will be provided to them.

## Discussion

SKY-D is expected to be the largest RCT to date examining the effectiveness of low-dose ketamine for young people with MDD. If the treatment proves to be effective, the trial will have significant clinical impact in terms of identifying a viable, rapidly acting antidepressant treatment option for young people with depression. SKY-D will also make a substantial contribution to knowledge about ketamine treatment for depression in relation to repeated dosing schedules, dosage titration, longer-term safety outcomes, mechanisms of action, and predictors of response.

The COVID-19 pandemic has had effects on SKY-D in terms of recruitment and its broader influence on young people’s mental health and will be an important consideration when the study is completed, the data analysed, and the results are published.

## Trial status

The protocol reported here is version 2.6 dated 2 February 2021. This trial is currently recruiting participants from all three sites. Recruitment for this study commenced in June 2019 and is expected to be completed in March 2024.

## Data Availability

All investigators will have access to the final dataset.

## Abbreviations

AE: Adverse event
AQoL-8D: Assessment of Quality of Life – 8 Dimensions
ASSIST: Alcohol, Smoking and Substance Involvement Screening Test
BPRS: Brief Psychiatric Rating Scale
BSCS: Brief Substance Craving Scale
CADSS: Clinician Administered Dissociative Symptoms Scale
CGI – S/I: Clinical Global Impression – Severity/Improvement
CPI: Co-ordinating principal investigator
CSD: Consensus Sleep Diary
C-SSRS: Columbia Suicide Severity Rating Scale
DSM-5: Diagnostic and Statistical Manual of Mental Disorders, 5th Edition
DEQ: Drug Effects Questionnaire
DSMB: Data safety monitoring board
eCRF: Electronic case report form
fMRI: Functional magnetic resonance imaging
GAD-7: Generalized Anxiety Disorder 7-item Scale
GCP: Good Clinical Practice
HMS: Hood Mysticism Scale
HREC: Human Research Ethics Committee
ICH: International Conference for Harmonisation
ITT: Intention to treat
KSET: Ketamine Side-Effects Tool
MADRS: Montgomery-Åsberg Depression Rating Scale
MDD: Major depressive disorder
MRI: Magnetic resonance imaging
NHMRC: National Health & Medical Research Council
NMDAR: N-methyl-d-aspartate receptor
PGI-I: Patient Global Impression – Improvement
PI: Principal investigator
PSQI: Pittsburgh Sleep Quality Inventory
QIDS: Quick Inventory of Depression Symptomatology
RCT: Randomised control trial
rMEQ: Revised Morningness-Eveningness Questionnaire
RNA: Ribonucleic acid
RPMS: Research Project Management System
SAE: Serious adverse event
SCID-5: Structured Clinical Interview for DSM-5
SIS: Suicidal Ideation Screen
SKY-D: Study of Ketamine for Youth Depression
SOFAS: Social and Occupational Functioning Assessment Scale
WHO: World Health Organization
